# Impact of the COVID-19 pandemic on reported cancer diagnoses in Bavaria, Germany

**DOI:** 10.1007/s00432-023-04707-0

**Published:** 2023-03-24

**Authors:** Sven Voigtländer, Amir Hakimhashemi, Nina Grundmann, Martin Radespiel-Tröger, Elisabeth C. Inwald, Olaf Ortmann, Michael Gerken, Stefanie J. Klug, Monika Klinkhammer-Schalke, Martin Meyer, Jacqueline Müller-Nordhorn

**Affiliations:** 1 Bavarian Cancer Registry, Bavarian Health and Food Safety Authority, Schweinauer Hauptstraße 80, 90441 Nuremberg, Germany; 2 Department of Gynecology and Obstetrics, University Medical Center, Landshuter Straße 65, 93053 Regensburg, Germany; 3 Institute for Quality Assurance and Health Services Research, University of Regensburg, Am BioPark 9, 93053 Regensburg, Germany; 4 Chair of Epidemiology, Department of Sport and Health Sciences, Technical University of Munich, Georg-Brauchle-Ring 56, 80992 Munich, Germany

**Keywords:** COVID-19, Pandemics, Cancer, Benign neoplasms, Incidence, Cancer registry

## Abstract

**Purpose:**

The aim of our study was to explore the impact of the COVID-19 pandemic on reported cancer cases in Bavaria, Germany, by comparing pre-pandemic (March 2019 to February 2020) and pandemic period (March 2020 to February 2021).

**Methods:**

Data on incident cases were retrieved from the Bavarian Cancer Registry (until 22nd April 2022). We included patients with malignant and in situ neoplasms reported by pathology departments with consistent reporting. We calculated the number of incident cases during the COVID-19 pandemic and the pre-pandemic period with 95% confidence intervals (CI) with Bonferroni correction (α = 0.0018) based on a Poisson approach. We stratified for malignancy (malignant, in situ), tumor site, and month of year.

**Results:**

Data was available for 30 out of 58 pathology departments (51.7%) from Bavaria. Incident malignant neoplasms dropped from 42,857 cases in the pre-pandemic period to 39,980 cases in the pandemic period (− 6.7%; 95% CI − 8.7%, − 4.7%). Reductions were higher for colon, rectum, skin/melanoma as well as liver (> 10.0% reduction) and less for breast cancer (4.9% reduction). No case reductions were observed for pancreas, esophagus, ovary, and cervix. Percent changes were largest for April 2020 (− 20.9%; 95% CI − 24.7%, − 16.8%) and January 2021 (− 25.2%; 95% CI − 28.8%, − 21.5%) compared to the previous year. Declines tended to be larger for in situ compared to malignant neoplasms.

**Conclusion:**

Detection and diagnosis of cancer were substantially reduced during the COVID-19 pandemic. Potential effects, e.g. a stage shift of tumors or an increase of cancer mortality, need to be monitored.

**Supplementary Information:**

The online version contains supplementary material available at 10.1007/s00432-023-04707-0.

## Introduction

The management of the Coronavirus disease 2019 (COVID-19) pandemic included far-reaching measures at the height of the pandemic, i.e. lockdowns and the postponement of elective surgery, with potential effects on the detection and diagnosis of cancer. Bavaria, Germany, experienced two lockdowns during the first year of the pandemic, the first lockdown from 21st March 2020 (Bavarian State Ministry of Health and Care 2020c) until 3rd May 2020 (Bavarian State Ministry of Health and Care 2020e) and the second lockdown from 9th December 2020 (Bavarian State Ministry of Health and Care 2020d) until 7th March 2021 (Bavarian State Ministry of Health and Care 2021). In addition, the Bavarian state suspended or postponed elective surgery and other planned procedures between 20th March 2020 and 15th May 2020 (Bavarian State Ministry of Health and Care 2020b). From 3rd November 2020 onwards the postponement of elective procedures was reintroduced in case of a local lack of hospital capacity (Bavarian State Ministry of Health and Care 2020a). In the whole of Germany, the organized mammography screening was suspended from 26th March to 30th April 2020 (Federal Joint Committee 2020), which was similar to other countries such as the Netherlands (Dinmohamed et al. 2020). Furthermore, measures taken by health care providers to respond to the COVID-19 pandemic (Alom et al. 2021) reduced treatment capacities and patients might have postponed diagnostic procedures for fear of infection (Stang et al. 2020). Apart from the organized mammography screening program (since 2009 in Bavaria), the German health care system has implemented screening programs for colorectal cancer (organized screening since 2019, prior to that opportunistic) and cervical cancer (organized screening since 2020, prior to that opportunistic), while opportunistic screening exists for skin cancer and prostate cancer, including reimbursement from the statutory health insurance (Federal Joint Committee (GBA), 2022). None of these activities—apart from mammography screening—has been officially suspended during the COVID-19 pandemic, however, screening may have been suspended or postponed due to reduced treatment capacities of health care providers.

Pandemic control measures may have led to diagnostic and treatment delays as well as de-escalation and modification of treatments (Alom et al. 2021; Bakouny et al. 2020), potentially resulting in negative long-term effects on cancer survival (Hanna et al. 2020; Sud et al. 2020). Hanna et al. (2020), for instance, estimated that the risk of death increased for each four week delay in surgery by 8% for breast cancer and 6% for colon cancer. Similar estimates were found for each four-week delay in adjuvant and neoadjuvant systemic treatment and the risk of death (Hanna et al. 2020). Previous studies showed a drop in screening rates (Chen et al. 2021; Dinmohamed et al. 2020; Teglia et al. 2022), cancer reporting by pathology departments (Johansson et al. 2022; Peacock et al. 2021; Ribes et al. 2022; Stang et al. 2020) as well as the number of patients with newly identified cancer (Angelini et al. 2023; Coma et al. 2021; Epidemiologisches Krebsregister Baden-Württemberg 2022; Kaufman et al. 2021; Morris et al. 2021; Voigtländer et al. 2021), and a delay of non-urgent/elective surgery (Bakouny et al. 2020) during the COVID-19 pandemic. Significant reductions observed for all major tumor sites including breast, prostate, colorectum, and lung (Chen et al. 2021; Coma et al. 2021; Johansson et al. 2022; Kaufman et al. 2021; Morris et al. 2021; Peacock et al. 2021; Ribes et al. 2022) were larger in the first COVID-19 wave around April and May 2020 compared to the second COVID-19 wave around November 2020, December 2020, and January 2021 (Coma et al. 2021; Johansson et al. 2022; Kaufman et al. 2021; Peacock et al. 2021; Ribes et al. 2022). Between the first and the second COVID-19 wave the number of patients diagnosed with cancer recovered, but did—in most countries—not reach pre-pandemic levels (Coma et al. 2021; Johansson et al. 2022; Kaufman et al. 2021; Morris et al. 2021; Peacock et al. 2021; Ribes et al. 2022; Voigtländer et al. 2021), so that many cancers may remain undiagnosed for a longer period of time (Kaufman et al. 2021).

Tumor sites differed regarding the observed reductions in screening as well as incident cancer cases. A meta-analysis on the global association of the COVID-19 pandemic measures with breast, colorectal as well as cervical cancer screening found that, although all three screenings had been heavily impacted by the COVID-19 pandemic, only colorectal cancer screening was significantly decreased after May 2020 (Teglia et al. 2022). Regarding incident cancer cases during the first COVID-19 wave, reductions were generally larger for melanoma, prostate, colorectum as well as breast compared to pancreas, lung, cervix, esophagus as well as bladder (Coma et al. 2021; Kaufman et al. 2021; Peacock et al. 2021; Ribes et al. 2022), which may be due to obvious symptoms and a poorer prognosis, for instance, for lung as well as pancreatic cancer (Peacock et al. 2021). One study from Germany found no reduction in lung cancer cases during the first COVID-19 wave (Epidemiologisches Krebsregister Baden-Württemberg 2022). Regarding differences between tumor sites, a reporting bias cannot be excluded as mentioned by Skovlund et al. (2022), who arrived at slightly different results based on two different dates of data extraction. The effects on cancer incidence seemed different during the second compared to the first COVID-19 wave, i.e. reductions were smaller during the second wave, especially for breast cancer (Coma et al. 2021; Kaufman et al. 2021; Peacock et al. 2021; Ribes et al. 2022).

Differences regarding malignant and in situ cases as well as differences according to age and sex have been investigated by few studies so far (Coma et al. 2021; Johansson et al. 2022; Peacock et al. 2021; Ribes et al. 2022). In a study comparing the Nordic countries, reductions have been larger for in situ cases compared to malignant cases (Johansson et al. 2022). In our previous study (Voigtländer et al. 2021), we found that reductions were larger for stage I tumors, however, we did not include in situ cases. Regarding age group and sex differences, the evidence is inconclusive (Epidemiologisches Krebsregister Baden-Württemberg 2022; Johansson et al. 2022; Peacock et al. 2021; Ribes et al. 2022).

The aim of our study was to explore the impact of the COVID-19 pandemic on the reported cancer cases in Bavaria, Germany, by comparing pandemic and pre-pandemic period with a special focus on the lockdown periods. We stratified by malignancy, tumor site, age, and sex. In this study, we focused on reports by pathology departments as this data is most rapidly available and comprises a relatively large number of cases.

## Materials and methods

### Data

Data on incident cases were retrieved from the Bavarian Cancer Registry (until 22nd April 2022). We included malignant neoplasms (International Statistical Classification of Diseases and Related Health Problems, Tenth Revision (ICD-10) codes C00-C97 without C44 and C77-C79) as well as carcinoma in situ (ICD-10 D00-D09 without D04) reported by pathology departments with consistent reporting and complete registration. ICD-10 coding of tumors into chapter C and chapter D is based on the tumor’s topographic and morphologic characteristics according to the International Classification of Diseases for Oncology, Third Edition (ICD-O3) (Fritz et al. 2013; World Health Organization & International Agency for Research on Cancer 2022). Assessment of consistent reporting was done by our six regional registration centers, who tracked reporting of pathology departments throughout the study period (1st March 2019 to 28th February 2021) and verified that all notifications of pathology departments with consistent reporting were completely documented in our cancer registry before data extraction. The number of included pathology departments was constant across the study period.

### Variables

We stratified by period (pre-pandemic period, 1st March 2019 to 29th February 2020; pandemic period, 1st March 2020 to 28th February 2021), malignancy (malignant, in situ), and tumor site according to the ICD-10 codes (Breast, C50, D05; Prostate, C61, D07.5; Colon, C18, D01.0; Lung, C34, D02.2; Bladder, C67, D09.0; Cervix uteri, C53, D06; Rectum, C19/C20, D01.1/D01.2; Stomach, C16, D00.2; Non-Hodgkin lymphoma, C82-C88, C96; Skin/Melanoma, C43, D03; Corpus uteri, C54/C55; Lip, oral cavity, and pharynx, C00-C14, D00.0; Kidney, C64; Pancreas, C25, D01.7; Ovary, C56; Esophagus, C15; Liver, C22, D01.5; Leukemia, C91-C95; Other) as well as month of year. Furthermore, we differentiated between age groups (0–49, 50–69, 70 years and above) and sexes (Male, Female).

### Statistical analyses

We calculated the number of incident cases in the year preceding the pandemic (March 2019 to February 2020) and during the first year of the COVID-19 pandemic (March 2020 to February 2021), stratified by month, with 95% confidence intervals (CI) based on a Poisson distribution. Relative changes were estimated based on a t-distribution with 95% CI. Analyses were stratified by malignancy, tumor site, month of year, age group, and sex. Due to the introduction of the organized cervical cancer screening in January 2020, the year when the COVID-19 pandemic started, we ran the stratified analyses (malignancy, month of year, age, and sex) with and without cervical cancer (malignant and in situ). Stratification for tumor site was limited to sites with at least 1000 incident cases across the study period and, regarding carcinoma in situ, to sites with at least 100 incident cases. Multiple testing was accounted for by Bonferroni correction (α error of 0.0018 with 28 significance tests) and calculation of a correspondingly adjusted CI. Regarding cervical cancer (malignant and in situ), we additionally performed an age-stratified analysis, considering the change to an organized screening program including the introduction of an additional human papillomavirus (HPV) test for women aged 35 years and above in January 2020 (Federal Joint Committee (GBA), 2022; Orumaa et al. 2019). Following Johansson et al. (2022), we additionally calculated cumulative percentage change during the pandemic period as the difference in monthly cumulative cases between pandemic and pre-pandemic period divided by the total number of cases at the end of the pre-pandemic period. Thus, cumulative percentage change indicates cumulative deviation at each month from the pre-pandemic period. CI for cumulative percentage change were derived by bootstrap resampling with 1,000,000 resamples. All statistical analyses were performed using R (R Foundation for Statistical Computing), version 4.1.1.

## Results

Data was available for 30 out of 58 pathology departments (51.7%) in Bavaria comprising pathology departments from all regions within Bavaria. Response was slightly lower for inpatient pathology departments (12 out of 26; 46.2%) compared to outpatient pathology departments (18 out of 32; 56.3%). Characteristics of the study sample are presented in Table 1. Of the 91,856 incident cases included, 46,937 (51.1%) were observed in the pre-pandemic period and 44,919 (48.9%) in the pandemic period. Majority of total cases occurred in middle and older age groups (50–69 years: 43.4%; 70 years and above: 43.8%) and were malignant (90.2%), while sex distribution was balanced (women: 51.0%; men: 49.0%). Most frequent tumor sites, including malignant neoplasms and carcinoma in situ, were breast with 18,657 cases (20.3%), prostate with 12,647 cases (13.8%), colon with 8056 cases (8.8%), and lung with 6844 cases (7.5%).

**Table 1:** Characteristics of incident cases reported by pathology departments in Bavaria from March 2019 to February 2021, by pre-pandemic period (March 2019 to February 2020) and pandemic period (March 2020 to February 2021)

Characteristic	Pre-pandemic period	Pandemic period	Total
Cases (n, %)	46,937 (51.1)	44,919 (48.9)	91,856 (100.0)
Sex			
Male (n, %)	23,470 (50.0)	21,564 (48.0)	45,034 (49.0)
Female (n, %)	23,461 (50.0)	23,350 (52.0)	46,811 (51.0)
Other (n, %)	6 (< 0.1)	5 (< 0.1)	11 (< 0.1)
Age			
0–49 years (n, %)	5506 (11.7)	6259 (13.9)	11,765 (12.8)
50–69 years (n, %)	20,544 (43.8)	19,356 (43.1)	39,900 (43.4)
70 years and above (n, %)	20,887 (44.5)	19,304 (43.0)	40,191 (43.8)
Mean age male (yrs, SD)	67.8 (12.7)	67.8 (12.6)	67.8 (12.7)
Median age male (yrs)	69	69	69
Mean age female (yrs, SD)	64.0 (15.4)	62.7 (15.9)	63.3 (15.6)
Median age female (yrs)	65	64	65
Malignancy			
Malignant (n, %)	42,857 (91.3)	39,980 (89.0)	82,837 (90.2)
In situ (n, %)	4080 (8.7)	4939 (11.0)	9019 (9.8)
Tumor site (ICD-10)			
Breast (C50, D05) (n, %)	9608 (20.5)	9049 (20.1)	18,657 (20.3)
Prostate (C61, D07.5) (n, %)	6662 (14.2)	5985 (13.3)	12,647 (13.8)
Colon (C18, D01.0) (n, %)	4315 (9.2)	3741 (8.3)	8056 (8.8)
Lung (C34, D02.2) (n, %)	3545 (7.6)	3299 (7.3)	6844 (7.5)
Bladder (C67, D09.0) (n, %)	2460 (5.2)	2428 (5.4)	4888 (5.3)
Cervix uteri (C53, D06) (n, %)	1505 (3.2)	2688 (6.0)	4193 (4.6)
Rectum (C19/C20, D01.1/D01.2) (n, %)	2194 (4.7)	1844 (4.1)	4038 (4.4)
Stomach (C16, D00.2) (n, %)	1458 (3.1)	1398 (3.1)	2856 (3.1)
Non-Hodgkin lymphoma (C82-C88, C96) (n, %)	1309 (2.8)	1307 (2.9)	2616 (2.9)
Skin/Melanoma (C43, D03) (n, %)	1392 (3.0)	1158 (2.6)	2550 (2.8)
Corpus uteri (C54/C55) (n, %)	1265 (2.7)	1167 (2.6)	2432 (2.7)
Lip, oral cavity, and pharynx (C00-C14, D00.0) (n, %)	1221 (2.6)	1128 (2.5)	2349 (2.6)
Kidney (C64) (n, %)	1197 (2.6)	1092 (2.4)	2289 (2.5)
Pancreas (C25, D01.7) (n, %)	1020 (2.2)	1052 (2.3)	2072 (2.3)
Ovary (C56) (n, %)	608 (1.3)	683 (1.5)	1291 (1.4)
Esophagus (C15) (n, %)	610 (1.3)	616 (1.4)	1226 (1.3)
Liver (C22, D01.5) (n, %)	639 (1.4)	561 (1.2)	1200 (1.3)
Leukemia (C91-C95) (n, %)	633 (1.3)	563 (1.3)	1196 (1.3)
Other (n, %)	5296 (11.3)	5160 (11.5)	10,456 (11.4)

*n* number, *yrs* years, *SD* standard deviation, *ICD-10* international statistical classification of diseases and related health problems, tenth revision

Incident malignant neoplasms dropped significantly from 42,857 cases in the pre-pandemic period to 39,980 cases in the pandemic period (− 6.7%; 95% CI − 8.7 to − 4.7%) (Table 2, upper part). Reductions in incident malignant neoplasms were more pronounced for colon (− 11.5%; 95% CI − 17.6 to − 4.9%), rectum (− 15.4%; 95% CI − 23.5 to − 6.5%), skin/melanoma (− 15.6%; 95% CI − 26.5 to − 3.0%) and less pronounced for breast (− 4.9%; 95% CI − 9.3 to − 0.2%). No significant changes were observed for malignant tumors of the remaining tumor sites such as bladder, non-Hodgkin lymphoma, pancreas, esophagus as well as cervix uteri and ovary. Cumulative percentage changes for breast, prostate, colon, lung, and rectal cancer are shown in Supplementary Fig. 1.

**Table 2:** Incident malignant neoplasms as well as incident carcinoma in situ in total and by tumor site during the COVID-19 pandemic (March 2020 to February 2021) compared to the pre-pandemic period (March 2019 to February 2020)

Tumor site (ICD-10)	Pre-pandemic period: cases	Pandemic period: cases	Difference (% [95% CI])^a^
Malignant neoplasms			
All sites (C00-C97 without C44 and C77-C79)	42,857	39,980	− 2877 (− 6.7 [− 8.7; − 4.7])*
Breast (C50)	8703	8278	− 425 (− 4.9 [− 9.3; − 0.2])*
Prostate (C61)	6649	5974	− 675 (− 10.2 [− 15.0; − 5.0])*
Colon (C18)	4032	3568	− 464 (− 11.5 [− 17.6; − 4.9])*
Lung (C34)	3536	3294	− 242 (− 6.8 [− 13.6; 0.5])
Rectum (C19/C20)	2095	1772	− 323 (− 15.4 [− 23.5; − 6.5])*
Stomach (C16)	1450	1393	− 57 (− 3.9 [− 14.5; 8.0])
Bladder (C67)	1359	1347	− 12 (− 0.9 [− 12.1; 11.8])
Non-Hodgkin lymphoma (C82-C88, C96)	1309	1307	− 2 (− 0.2 [− 11.6; 12.8])
Corpus uteri (C54/C55)	1265	1167	− 98 (− 7.7 [− 18.7; 4.7])
Lip, oral cavity, and pharynx (C00-C14)	1197	1110	− 87 (− 7.3 [− 18.6; 5.6])
Kidney (C64)	1197	1092	− 105 (− 8.8 [− 19.9; 4.0])
Skin/Melanoma (C43)	1112	939	− 173 (− 15.6 [− 26.5; − 3.0])*
Pancreas (C25)	999	1039	40 (4.0 [− 9.4; 19.4])
Leukemia (C91-C95)	633	563	− 70 (− 11.1 [− 25.8; 6.6])
Liver (C22)	630	554	− 76 (− 12.1 [− 26.7; 5.5])
Esophagus (C15)	610	616	6 (1.0 [− 15.5; 20.7])
Ovary (C56)	608	683	75 (12.3 [− 5.6; 33.7])
Cervix uteri (C53)	463	508	45 (9.7 [− 10.2; 34.1])
Other	5010	4,776	− 234 (− 4.7 [− 10.5; 1.5])
Carcinoma in situ			
All sites (D00-D09 without D04)	4080	4939	859 (21.1 [13.3; 29.3])*
Bladder (D09.0)	1101	1081	− 20 (− 1.8 [− 14.1; 12.2])
Cervix uteri (D06)	1042	2180	1,138 (109.2 [86.0; 135.3])*
Breast (D05)	905	771	− 134 (− 14.8 [− 26.9; − 0.7])*
Colon (D01.0)	283	173	− 110 (− 38.9 [− 54.8; − 17.4])*
Skin/Melanoma (D03)	280	219	− 61 (− 21.8 [− 41.0; 3.7])
Rectum (D01.1/D01.2)	99	72	− 27 (− 27.3 [− 55.2; 17.9])
Other	370	443	73 (19.7% [− 3.9%; 49.2%])

*ICD-10* international statistical classification of diseases and related health problems, tenth revision, *CI* confidence interval

*Indicates that the relative change is significantly different from zero with a significance level of 5% with Bonferroni correction (effective α error of 0.18%)

^a^CI with Bonferroni correction (effective CI of 99.82%)

For the total of carcinoma in situ, incident cases increased significantly from 4080 to 4939 cases (21.1%; 95% CI 13.3–29.3%) (Table 2, lower part). This increase was due to a large increase in cervical carcinoma in situ (109.2%; 95% CI 86.0–135.3%). Colon (− 38.9%; 95% CI − 54.8 to − 17.4%) as well as breast (− 14.8%; 95% CI − 26.9 to − 0.7%) decreased significantly. Rectum, skin/melanoma, and bladder did not decrease significantly. The age-stratified analysis for cervical carcinoma in situ showed that the percentage changes were higher for women aged 35 years and above (158.2%; 95% CI 133.8–185.2%) compared to women aged 20–34 years (56.4%; 95% CI 39.8–75.0%) (Supplementary Table 1). Cumulative percentage changes for carcinoma in situ of the bladder, the cervix uteri, the breast, the colon, and skin/melanoma are shown in Supplementary Fig. 2.

**Fig. 1:**
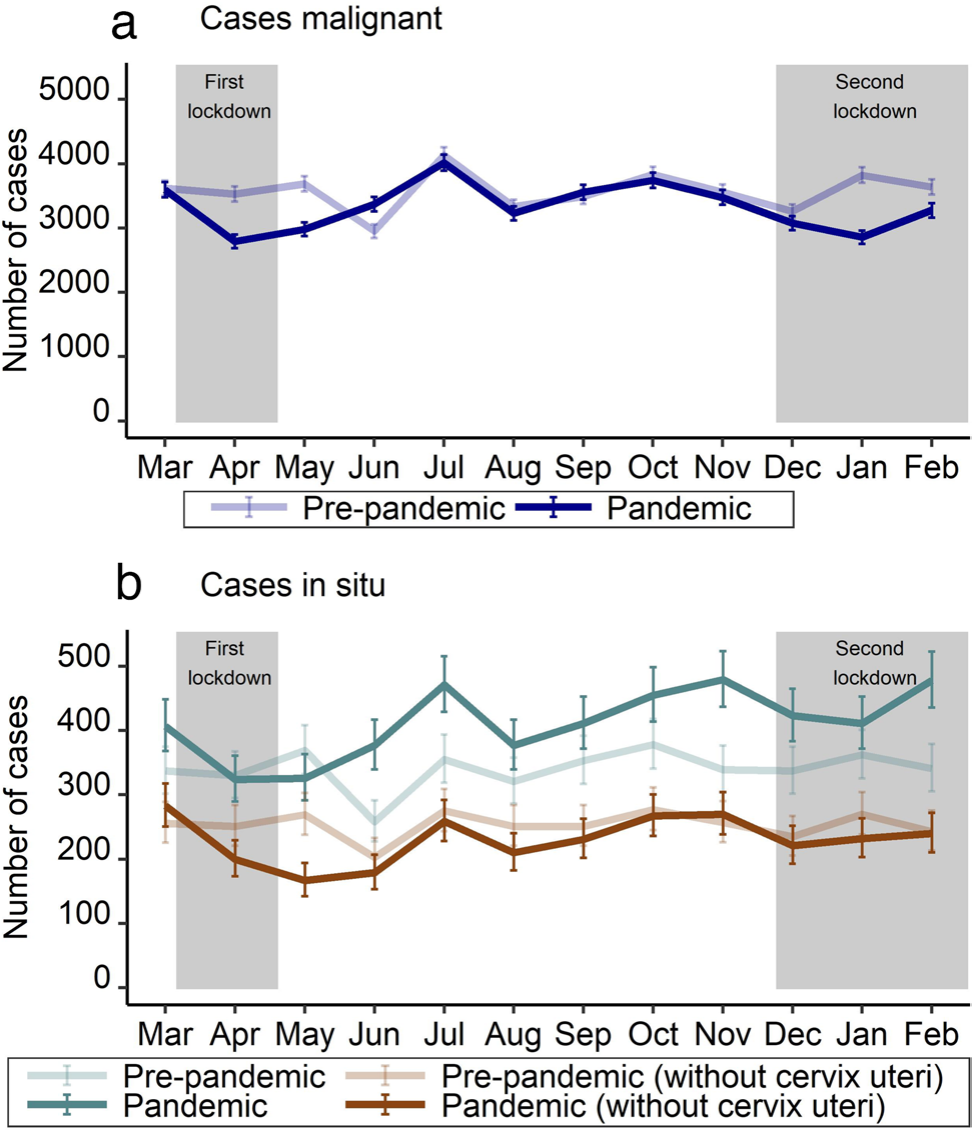
Number of incident malignant neoplasms (**a**) and carcinoma in situ (with and without cervix uteri) (**b**) from March 2019 to February 2021, stratified by period (pre-pandemic: March 2019 to February 2020; pandemic: March 2020 to February 2021) and month of year

**Fig. 2:**
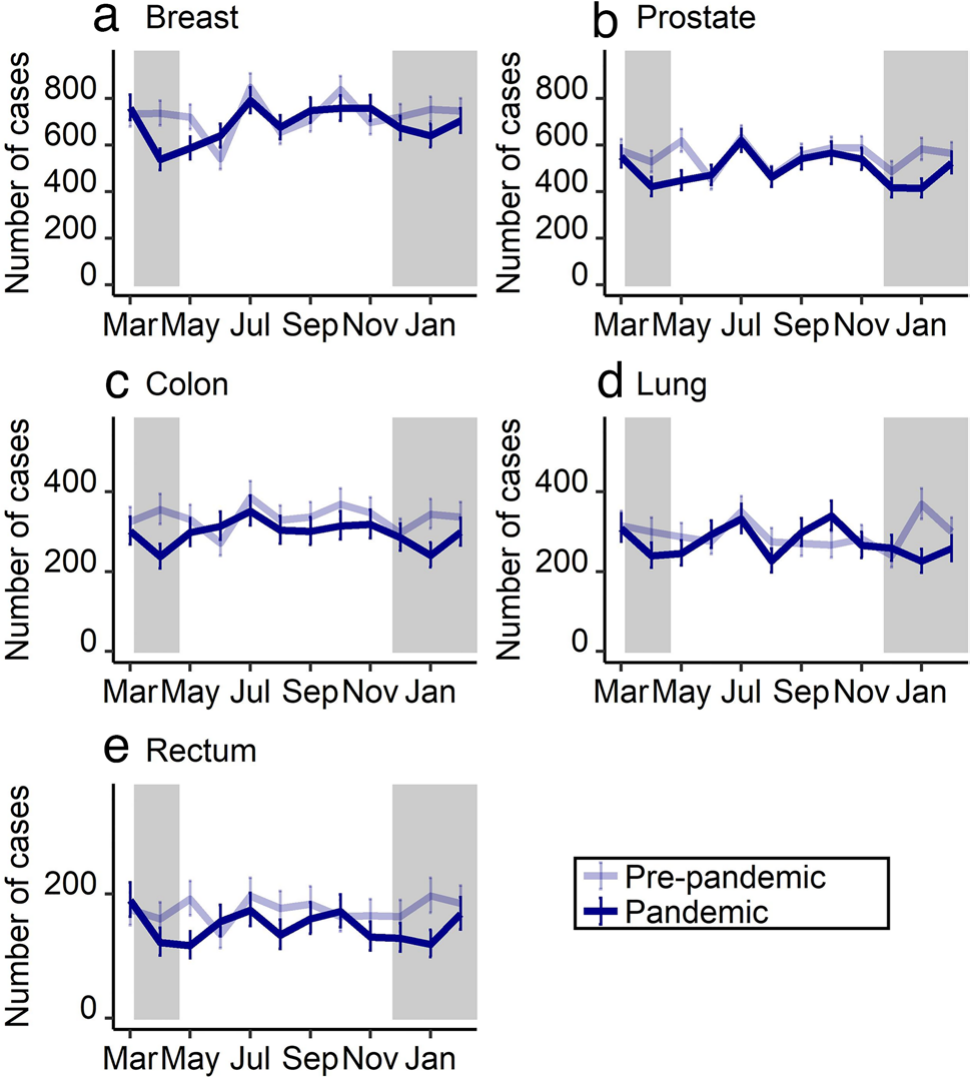
Number of incident malignant neoplasms for the five most frequent tumor sites [breast (**a**), prostate (**b**), colon (**c**), lung (**d**), and rectum (**e**)] from March 2019 to February 2021, stratified by period (pre-pandemic: March 2019 to February 2020; pandemic: March 2020 to February 2021) and month of year

**Fig. 3:**
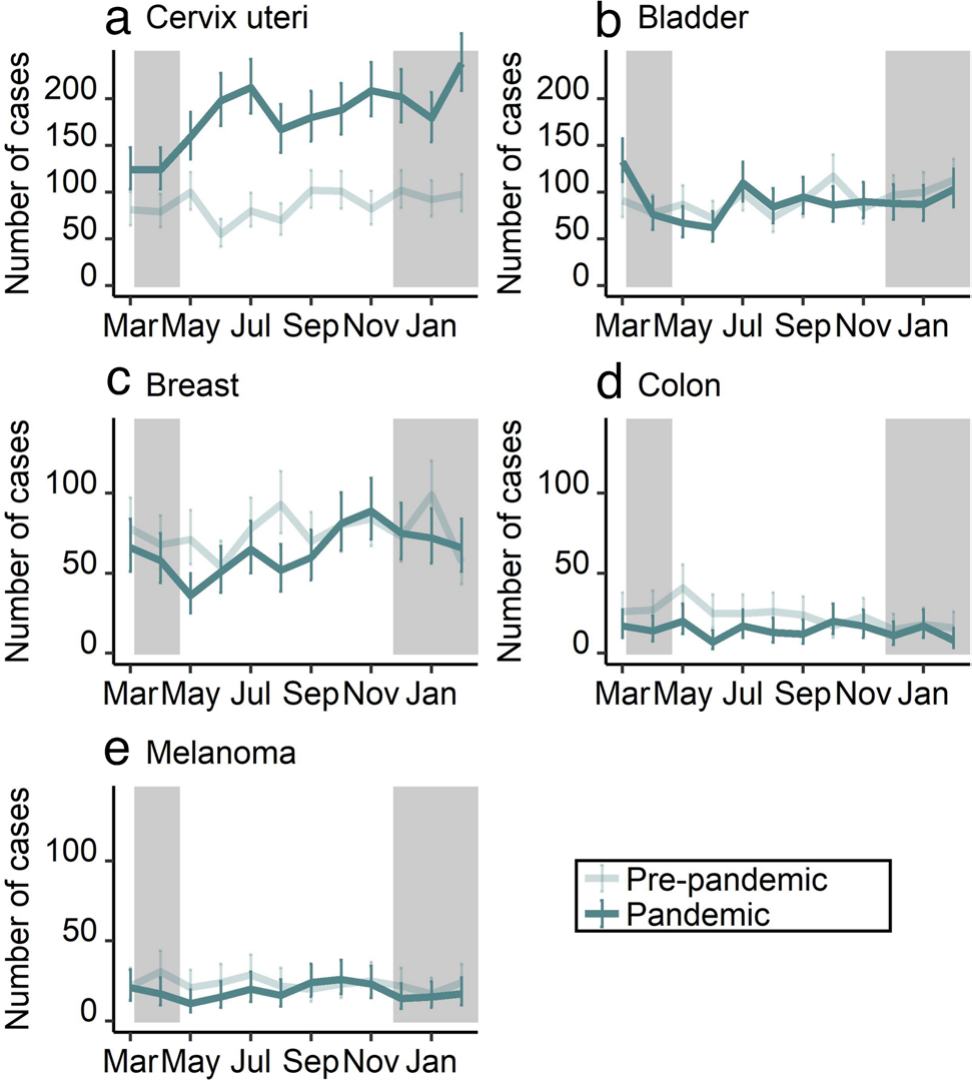
Number of incident carcinoma in situ for the five most frequent tumor sites [cervix uteri (**a**), bladder (**b**), breast (**c**), colon (**d**), and melanoma (**e**)] from March 2019 to February 2021, stratified by period (pre-pandemic: March 2019 to February 2020; pandemic: March 2020 to February 2021) and month of year

Stratified by month, the largest and significant declines in malignant neoplasms were observed during the two lockdowns, i.e. April 2020 (− 20.9%; 95% CI − 24.7 to − 16.8%), May 2020 (− 19.2%; 95% CI − 23.0 to − 15.2%), and January 2021 (− 25.2%; 95% CI − 28.8 to − 21.5%) compared to the same month of the previous year, respectively (Fig. 1, Supplementary Table 2). For June 2020, there was a significant increase by 14.2% (95% CI 8.6–19.9%). Carcinoma in situ showed similar trends like malignant tumors except for cervix uteri. Figure 2 and Fig. 3 show the trend in monthly number of cases for the most frequent tumor sites of malignant neoplasms and carcinoma in situ, respectively (see numbers in Supplementary Table 3 and Supplementary Table 4). For instance, the decline for malignant neoplasms of the breast was above the total average for all cancer cases in April 2020 with − 27.0% (95% CI − 34.7 to − 18.4%) but not in May 2020 (− 18.5%; 95% CI − 26.9 to − 9.1%) and January 2021 (− 15.0%; 95% CI − 23.5 to − 5.6%), each compared to the same month of the previous year. In June 2020, the increase was above average with 18.1% (95% CI 5.3–32.4%) compared to the previous year.

**Fig. 4:**
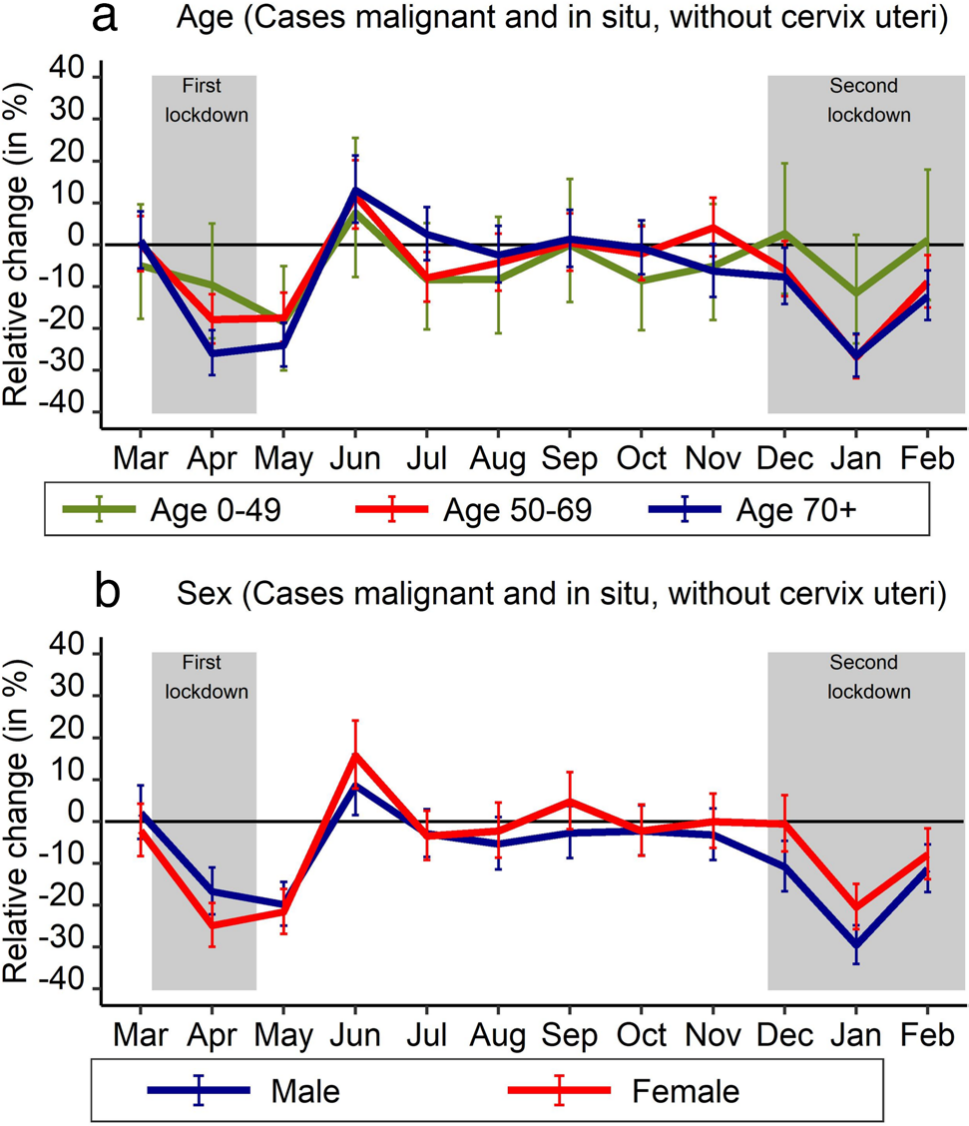
Number of incident malignant neoplasms (without cervical cancer) and carcinoma in situ (without cervical carcinoma in situ) according to age (**a**) and sex (**b**), stratified by period (pre-pandemic: March 2019 to February 2020; pandemic: March 2020 to February 2021) and month of year

Age group as well as sex stratification were performed for all incident cases (malignant and in situ, without cervix uteri) (Fig. 4, Supplementary Table 5, Supplementary Table 6). The number of reported cases was significantly reduced for all age groups. The size of the percent change increased with age (0–49 years: − 5.6%; 95% CI − 9.6 to − 1.6%; 50–69 years: − 6.6%; 95% CI − 8.5 to − 4.8%; 70 years and above: − 7.7%; 95% CI − 9.5 to − 5.9%), though differences between age groups were not significant. In April and May 2020, reductions were highest for those aged 70 years and above, while in January and February 2021, reductions were similar for the age groups 50–69 years and 70 years and above, each compared to the same month of the previous year. For the age group 0–49 years, the only significant monthly percent change was observed for May 2020, when it decreased compared to the previous year. Regarding sex, number of cases decreased stronger for men (− 8.1%; 95% CI − 9.8% to − 6.4%) than for women (− 5.9%; 95% CI − 7.7 to − 4.1%) during the pandemic period compared to the pre-pandemic period, though this difference was not significant. Percent decrease was higher for women than for men during the first lockdown, while it was the opposite during the second lockdown.

## Discussion

This study showed that the diagnosis of malignant neoplasms was significantly reduced during the COVID-19 pandemic. Between March 2020 and February 2021, 2877 fewer cases (− 6.7%) had been diagnosed as compared to the pre-pandemic period. Reductions were more pronounced for colon, rectum as well as skin/melanoma and less for breast. The number of malignant neoplasms did not decrease for pancreas, esophagus, ovary, and cervix uteri. Carcinoma in situ had similar trends like malignant tumors except for cervix uteri, which significantly increased. Stratified by month, reduction of incident cases was highest for lockdown months, i.e. April and May 2020 as well as January 2021. The size of the reductions increased with age and was higher for men than for women, though differences were not significant. For women, case reductions seemed to be slightly higher during the first COVID-19 lockdown and lower during the second COVID-19 lockdown compared to men.

The substantial decline in newly diagnosed cancer cases during the COVID-19 pandemic is in line with previous studies (Chen et al. 2021; Coma et al. 2021; Dinmohamed et al. 2020; Johansson et al. 2022; Kaufman et al. 2021; Morris et al. 2021; Peacock et al. 2021; Ribes et al. 2022; Teglia et al. 2022) including studies from Germany (Epidemiologisches Krebsregister Baden-Württemberg 2022; Voigtländer et al. 2021). Studies from Germany that were based on the number of notifications by pathology departments as well as by clinicians also support this finding (Justenhoven and Rieger 2022; Piontek et al. 2021; Stang et al. 2020). Similar to other studies, the decline was significant for most major tumor sites, such as breast, prostate, and colorectum (Chen et al. 2021; Coma et al. 2021; Epidemiologisches Krebsregister Baden-Württemberg 2022; Johansson et al. 2022; Kaufman et al. 2021; Morris et al. 2021; Peacock et al. 2021; Ribes et al. 2022). For lung cancer, however, we did not find a significant decline, as shown by other studies (Epidemiologisches Krebsregister Baden-Württemberg 2022). Stratified by month, incident cancer cases were not only reduced during the first COVID-19 wave but also during the second wave. The finding of similar reductions during the first and second wave is contrary to other studies (Coma et al. 2021; Johansson et al. 2022; Kaufman et al. 2021; Peacock et al. 2021; Ribes et al. 2022), which observed smaller reductions during the second compared to the first wave. However, these studies investigated regions and countries like, for instance, Catalonia and Belgium, which experienced much larger reductions of incident cancer cases of up to 40% during the first COVID-19 wave. Belgium, for instance, also had substantially higher rates of hospital admissions for COVID-19 (Our World in Data 2023b) as well as of COVID-19 patients in intensive care units (Our World in Data 2023a) during the first wave than Germany. The higher hospital rates for COVID-19 in Belgium may have led to a reduced number of cancer patients being admitted, as compared to Germany. As far as we know, none of the published studies from Germany so far studied the second COVID-19 wave. The studies either ended 2020 (Epidemiologisches Krebsregister Baden-Württemberg 2022; Piontek et al. 2021; Stang et al. 2020) or reported annual estimates (Justenhoven & Rieger 2022). Similar to other studies (Coma et al. 2021; Johansson et al. 2022; Kaufman et al. 2021; Morris et al. 2021; Peacock et al. 2021; Ribes et al. 2022; Voigtländer et al. 2021), we observed that the number of patients diagnosed with cancer recovered between the two COVID-19 lockdowns, but it did not compensate for the large drop in cancer cases, especially in April and May 2020 as well as in January 2021. Thus, pre-pandemic levels have not been reached, so that it is likely that many cancers remain undiagnosed for a longer period of time (Kaufman et al. 2021). Some cancers may have remained undiagnosed as patients may have died from COVID-19 before a cancer diagnosis could have been made. However, this effect seems to be small as excess mortality from COVID-19 was low in Germany compared to other European countries, i.e. Spain and Belgium (Islam et al. 2021; Kowall et al. 2021).

Tumor sites differed regarding the impact of the COVID-19 pandemic. As reported by previous studies for the first COVID-19 wave, we found larger case reductions for skin/melanoma, colon, rectum, prostate as well as breast and smaller or no reductions, respectively, for ovary, cervix uteri, pancreas, esophagus as well as bladder (Coma et al. 2021; Kaufman et al. 2021; Peacock et al. 2021; Ribes et al. 2022). Similar to these studies (Coma et al. 2021; Kaufman et al. 2021; Peacock et al. 2021; Ribes et al. 2022), declines have been smaller for breast cancer cases during the second COVID-19 wave. This might reflect the fact that the organized mammography screening had been suspended in the first but not in the second COVID-19 lockdown (Federal Joint Committee 2020).

Cancers, for which screening measures have been implemented, showed different trends during the COVID-19 pandemic. While skin/melanoma, rectum, colon, and prostate have been heavily impacted with above average reductions in the number of patients newly diagnosed with cancer, the reduction of breast cancer cases was, as already mentioned above, primarily associated with the first COVID-19 lockdown. For all of these screening sites (skin/melanoma, rectum, colon, prostate, and breast), however, we found, similar to previous studies, larger reductions for in situ cases compared to malignant cases (Johansson et al. 2022), especially colon and rectum (Teglia et al. 2022). This may reflect the decreased number of diagnostic services including screenings tests, which has been observed nationally (DAK-Gesundheit 2021; Tillmanns et al. 2022) and internationally (Chen et al. 2021; Dinmohamed et al. 2020; Morris et al. 2021; Teglia et al. 2022). Though the suspension of the mammography screening program has certainly played a role in the German context, the higher reductions for the other screening-related cancers suggest that other factors, such as measures taken by health care providers to respond to the COVID-19 pandemic including reduced treatment capacities (Alom et al. 2021) as well as the fear of infection (Stang et al. 2020) played a larger role. Reduced treatment capacities may be regarded as a “silent” suspension of screening or, more generally phrased, diagnostic activities. The Federal Statistical Office of Germany (2023), for instance, estimated that the number of in-patient cancer treatments fell from 1.56 million in the year 2019 to 1.45 million in 2020 and 1.44 million in 2021, respectively, which corresponds to a relative reduction of 6.1% from 2019 to 2020 and 1.2% from 2020 to 2021.

For the tumor site cervix uteri, we observed, in contrast to other studies (Ferrara et al. 2022), a large increase in newly diagnosed cases during the COVID-19 pandemic as compared to the pre-pandemic period, especially for carcinoma in situ with a significant doubling of cases. Several factors may explain this finding. One likely factor may be the implementation of an organized screening program in January 2020 (Federal Joint Committee (GBA), 2022; Orumaa et al. 2019), which preceded the first COVID-19 wave. The organized screening program replaced the previously opportunistic screening and included an additional HPV test for women aged 35 years and above. This hypothesis is substantiated by the age-stratified analysis, which found a higher increase in incident cases for women aged 35 years and above compared to women aged 20–34 years. In addition, the screening population for cervical cancer is relatively young compared to the other screenings and may have been less impacted by the COVID-19 pandemic. Other factors may be classification changes for cervical intraepithelial lesions in the ICD-O3 (Fritz et al. 2013; World Health Organization & International Agency for Research on Cancer 2022) in 2020 as well as an increasing focus of German cancer registries on the documentation of such lesions.

Age- and sex-specific differences were similar comparing the pandemic period to the pre-pandemic period, which is similar to a study from another German cancer registry (Epidemiologisches Krebsregister Baden-Württemberg 2022). A study from Belgium, for instance, found strong declines in the screening age groups for female breast cancer as well as for colorectal cancer (Peacock et al. 2021). Our analyses, stratified by month, point to a higher reduction in the number of cancer cases among those 70 years of age and above during the first COVID-19 wave, which may be explained by the higher risk of a severe COVID-19 disease. While we found higher case reductions during the first COVID-19 wave for women, probably due to the suspension of the breast cancer screening, the reductions were generally larger for men from August 2020 onwards. Similar to this, a study from Catalonia found that the COVID-19 pandemic had less impact on women than on men because of the early recovery of diagnoses of breast and gynecological tumors while the incidence of colorectal and tobacco-related cancers remained low (Ribes et al. 2022).

This study has several strengths and limitations. Strengths are the large database with more than 90,000 incident cases for a populous German federal state with more than 13 million inhabitants, which is higher than the population of countries like Belgium, Sweden or Portugal, as well as the stratification by tumor site, age, sex, and the study period covering the first and second wave of the COVID-19 pandemic.

A major limitation of this study is that we cannot exclude a selection bias with regard to the pathology departments in our study. Although we included half of all pathology departments in Bavaria, the pathology departments with consistent reporting may differ from the remaining pathology departments. This may lead to both, an over- or underestimation of the number of newly diagnosed cancer cases in Bavaria. Further limitations are that pathological data may not be directly comparable to official incidence, which takes into account additional notifications by clinicians. Pathological data does not include information on stage at diagnosis according to Union for International Cancer Control (UICC) (Brierley et al. 2016; Ribes et al. 2022). The assumption of stable underlying incidence rates across the year preceding the pandemic and the first year of the COVID-19 pandemic seems reasonable for a short study period (Johansson et al. 2022). Although we only included pathology departments with consistent reporting and complete registration, which was tracked by our six regional registration centers, we cannot rule out that COVID-19 mitigation efforts, e.g. staff quarantine and/or segregation systems (Alom et al. 2021), affected the cancer reporting to the Bavarian Cancer Registry (Skovlund et al. 2022).

In conclusion, detection and diagnosis of cancer were substantially reduced during the COVID-19 pandemic, especially during lockdown months. Reductions were highest for colon, rectum, skin/melanoma, and tended to be larger for carcinoma in situ compared to malignant neoplasms. The exception was cervical carcinoma in situ, which likely increased due to the implementation of an organized screening program before the COVID-19 pandemic as well as to classification changes for cervical intraepithelial neoplasia. Potential effects of reduced and delayed detection as well as diagnosis of cancer, e.g. stage shift and increased cancer mortality, need to be monitored.

## Supplementary Information

Below is the link to the electronic supplementary material.

Supplementary file1 (PDF 635 KB)

## Data Availability

Following publication, the anonymized datasets generated and/or analyzed during the current study are available from the corresponding author to researchers who provide a methodologically sound proposal, provided approval from the Advisory Board of the Bavarian Cancer Registry.
