# Supplementary material for: Impact of the COVID-19 pandemic on reported cancer diagnoses in Bavaria, Germany
Source: J Cancer Res Clin Oncol. 2023 Mar 24;149(10):7493–503. doi: 10.1007/s00432-023-04707-0 (PMC10038367; doi:10.1007/s00432-023-04707-0)
Supplement: Supplementary file 1 — Supplementary file1 (PDF 635 KB) [file 432_2023_4707_MOESM1_ESM.pdf]

Title:

Impact of the COVID-19 pandemic on reported cancer diagnoses in Bavaria, Germany

Journal:

Journal of Cancer Research and Clinial Oncology

Authors:

Sven Voigtländer[1], Amir Hakimhashemi[1], Nina Grundmann[1], Martin Radespiel-Tröger[1], Elisabeth C. Inwald[2], Olaf Ortmann[2], Michael Gerken[3], Stefanie J. Klug[4], Monika Klinkhammer-Schalke[3], Martin Meyer[1], Jacqueline Müller-Nordhorn[1]

[1] Bavarian Cancer Registry, Bavarian Health and Food Safety Authority, Schweinauer Hauptstraße 80, 90441 Nuremberg, Germany

[2] Department of Gynecology and Obstetrics, University Medical Center, Landshuter Straße 65, 93053 Regensburg

[3] Institute for Quality Assurance and Health Services Research, University of Regensburg, Am BioPark 9, 93053 Regensburg

[4] Chair of Epidemiology, Department of Sport and Health Sciences, Technical University of Munich, Georg-Brauchle-Ring 56, 80992 München

Corresponding author:

Dr. Sven Voigtländer

Bavarian Cancer Registry, Bavarian Health and Food Safety Authority

Schweinauer Hauptstraße 80, 90441 Nuremberg, Germany

Phone: +49-(0)9131-6808-2927

Email: sven.voigtlaender@lgl.bayern.de

ORCID ID: https://orcid.org/0000-0003-3808-9605

Supplementary Figures

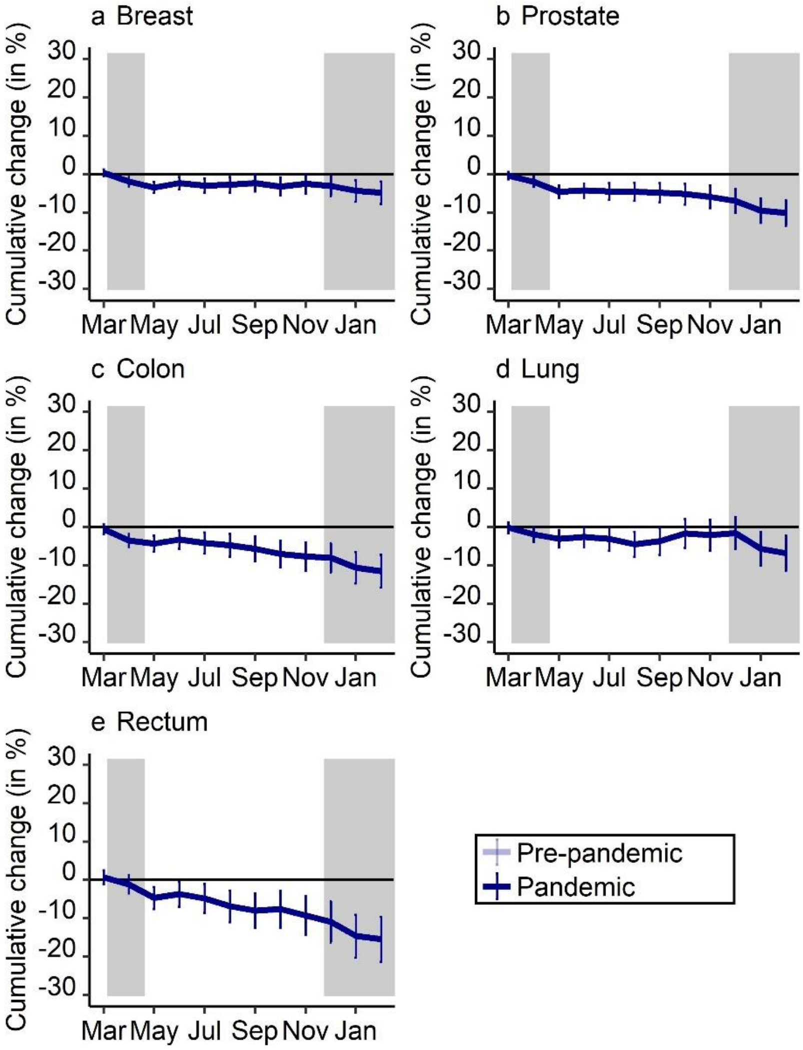

Supplementary Fig. 1 Cumulative percentage change of incident malignant neoplasms for the five most frequent sites (breast, prostate, colon, lung, and rectum) during the COVID-19 pandemic (March 2020 to February 2021) compared to the pre-pandemic period (March 2019 to February 2020), stratified by month of year

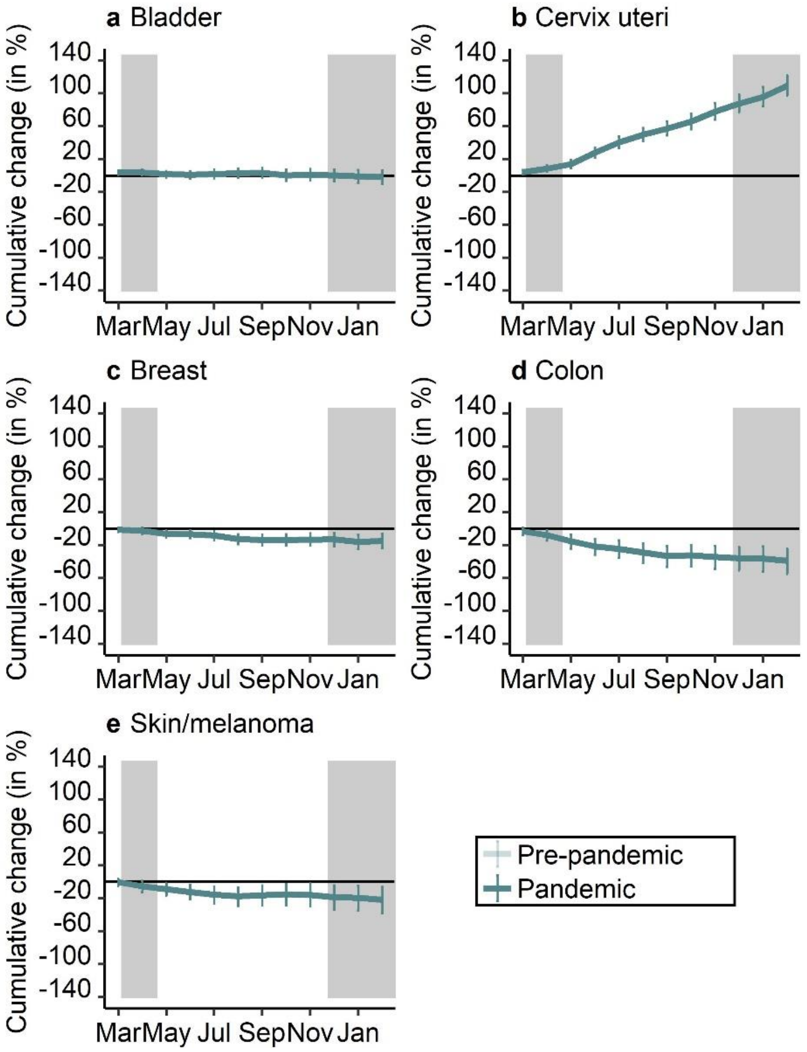

Supplementary Fig. 2 Cumulative percentage change of incident carcinoma in situ for the five most frequent sites (bladder, cervix uteri, breast, colon, and skin/melanoma) during the COVID-19 pandemic (March 2020 to February 2021) compared to the pre-pandemic period (March 2019 to February 2020), stratified by month of year

Supplementary Tables

Supplementary Table 1: Incident malignant neoplasms and carcinoma in situ for cervix uteri during the COVID-19 pandemic (March 2020 to February 2021) compared to the pre-pandemic period (March 2019 to February 2020)

Tumour site (ICD-10)	Pre-pandemic period: Cases	Pandemic period: Cases	Difference (% [95% CI])
**Cervix uteri (C53)**			
20-34 years	51	51	0 (0.0 [-32.2; 47.4])
35 years and above	412	457	45 (10.9 [-2.9; 26.7])
Total	463	508	45 (9.7 [-3.3; 24.4])
**Cervix uteri (D06)**			
20-34 years	498	779	281 (56.4 [39.8; 75.0])
35 years and above	541	1,397	856 (158.2 [133.8; 185.2])
Total	1,042	2,180	1,138 (109.2 [94.3; 125.2])

Notes: ICD-10, International Statistical Classification of Diseases and Related Health Problems, Tenth Revision; CI, confidence interval.

Supplementary Table 2: Number of incident malignant neoplasms and carcinoma in situ (with and without cervix uteri) from March 2019 to February 2021, stratified by period (pre-pandemic: March 2019 to February 2020; pandemic: March 2020 to February 2021) and month of year

	**Pre-pandemic period: Cases**	**Pandemic period: Cases**	**Difference (% [95% CI])**
**Malignant Neoplasms**			
March	3,622	3,597	-25 (-0.7 [-5.2; 4.0])
April	3,529	2,793	-736 (-20.9 [-24.7; -16.8])
May	3,689	2,982	-707 (-19.2 [-23.0; -15.2])
June	2,954	3,372	418 (14.2 [8.6; 19.9])
July	4,130	4,018	-112 (-2.7 [-6.8; 1.6])
August	3,331	3,229	-102 (-3.1 [-7.6; 1.7])
September	3,490	3,558	68 (1.9 [-2.7; 6.8])
October	3,833	3,742	-91 (-2.4 [-6.7; 2.1])
November	3,560	3,476	-84 (-2.4 [-6.8; 2.3])
December	3,258	3,078	-180 (-5.5 [-10.1; -0.8])
January	3,823	2,858	-965 (-25.2 [-28.8; -21.5])
February	3,638	3,277	-361 (-9.9 [-14.1; -5.6])
Total	42,857	39,980	-2,877 (-6.7 [-8.0; -5.4])
**Carcinoma in situ (with cervix uteri)**			
March	337	407	70 (20.8 [4.5; 39.5])
April	330	324	-6 (-1.8 [-15.8; 14.4])
May	369	326	-43 (-11.7 [-23.9; 2.5])
June	258	377	119 (46.1 [24.7; 71.2])
July	355	471	116 (32.7 [15.6; 52.3])
August	321	377	56 (17.4 [1.2; 36.3])
September	353	411	58 (16.4 [1.0; 34.2])
October	378	455	77 (20.4 [5.0; 38.0])
November	339	479	140 (41.3 [22.9; 62.4])
December	337	423	86 (25.5 [8.8; 44.8])
January	362	411	49 (13.5 [-1.4; 30.8])
February	341	478	137 (40.2 [22.0; 61.1])
Total	4,080	4,939	859 (21.1 [16.1; 26.2])
**Carcinoma in situ (without cervix uteri)**			
March	256	283	27 (10.5 [-6.6; 30.9])
April	251	200	-51 (-20.3 [-33.8; -4.1])
May	269	167	-102 (-37.9 [-48.8; -24.7])
June	203	179	-24 (-11.8 [-27.9; 7.8])
July	275	259	-16 (-5.8 [-20.5; 11.6])
August	251	210	-41 (-16.3 [-30.3; 0.5])
September	251	231	-20 (-8.0 [-23.0; 10.0])
October	277	267	-10 (-3.6 [-18.5; 14.0])
November	257	270	13 (5.1 [-11.4; 24.6])
December	235	221	-14 (-6.0 [-21.7; 13.0])
January	270	232	-38 (-14.1 [-27.9; 2.4])
February	243	240	-3 (-1.2 [-17.4; 18.1])
Total	3,038	2,759	-279 (-9.2 [-13.7; -4.4])

Notes: CI, confidence interval.

Supplementary Table 3: Number of incident malignant neoplasms from March 2019 to February 2021, stratified by tumour site (breast, prostate, colon, lung, rectum), period (pre-pandemic: March 2019 to February 2020; pandemic: March 2020 to February 2021) and month of year

	Pre-pandemic period: Cases	Pandemic period: Cases	Difference (% [95% CI])
**Breast**			
March	733	761	28 (3.8 [-6.2; 14.9])
April	737	538	-199 (-27.0 [-34.7; -18.4])
May	720	587	-133 (-18.5 [-26.9; -9.1])
June	542	640	98 (18.1 [5.3; 32.4])
July	849	792	-57 (-6.7 [-15.3; 2.8])
August	655	676	21 (3.2 [-7.3; 14.9])
September	709	749	40 (5.6 [-4.7; 17.1])
October	839	758	-81 (-9.7 [-18.1; -0.3])
November	697	759	62 (8.9 [-1.7; 20.7])
December	722	673	-49 (-6.8 [-16.1; 3.5])
January	753	640	-113 (-15.0 [-23.5; -5.6])
February	747	705	-42 (-5.6 [-14.9; 4.6])
Total	8,703	8,278	-425 (-4.9 [-7.7; -2.0])
**Prostate**			
March	577	550	-27 (-4.7 [-15.2; 7.1])
April	529	421	-108 (-20.4 [-30.0; -9.5])
May	619	448	-171 (-27.6 [-35.9; -18.3])
June	451	471	20 (4.4 [-8.2; 18.8])
July	634	620	-14 (-2.2 [-12.5; 9.2])
August	468	462	-6 (-1.3 [-13.2; 12.3])
September	559	541	-18 (-3.2 [-14.0; 8.9])
October	588	566	-22 (-3.7 [-14.2; 8.0])
November	589	540	-49 (-8.3 [-18.4; 3.0])
December	487	416	-71 (-14.6 [-25.1; -2.6])
January	583	415	-168 (-28.8 [-37.2; -19.3])
February	565	524	-41 (-7.3 [-17.7; 4.4])
Total	6,649	5,974	-675 (-10.2 [-13.2; -7.0])
**Colon**			
March	325	302	-23 (-7.1 [-20.6; 8.7])
April	356	238	-118 (-33.1 [-43.3; -21.2])
May	331	298	-33 (-10.0 [-23.0; 5.3])
June	272	314	42 (15.4 [-1.9; 35.8])
July	387	352	-35 (-9.0 [-21.3; 5.1])
August	329	304	-25 (-7.6 [-20.9; 8.0])
September	337	301	-36 (-10.7 [-23.5; 4.3])
October	369	315	-54 (-14.6 [-26.6; -0.8])
November	348	319	-29 (-8.3 [-21.3; 6.7])
December	297	285	-12 (-4.0 [-18.4; 12.9])
January	344	241	-103 (-29.9 [-40.6; -17.4])
February	337	299	-38 (-11.3 [-24.1; 3.7])
Total	4,032	3,568	-464 (-11.5 [-15.4; -7.4])
**Lung**			
March	316	309	-7 (-2.2 [-16.4; 14.4])
April	300	240	-60 (-20.0 [-32.5; -5.2])
May	287	246	-41 (-14.3 [-27.7; 1.6])
June	276	293	17 (6.2 [-9.9; 25.1])
July	351	332	-19 (-5.4 [-18.6; 9.9])
August	275	227	-48 (-17.5 [-30.8; -1.6])
September	271	298	27 (10.0 [-6.7; 29.6])
October	267	340	73 (27.3 [8.5; 49.5])

November	283	266	-17 (-6.0 [-20.5; 11.1])
December	241	259	18 (7.5 [-9.8; 28.1])
January	369	226	-143 (-38.8 [-48.1; -27.7])
February	300	258	-42 (-14.0 [-27.2; 1.6])
Total	3,536	3,294	-242 (-6.8 [-11.2; -2.3])
**Rectum**			
March	175	190	15 (8.6 [-11.6; 33.3])
April	160	122	-38 (-23.8 [-39.8; -3.5])
May	192	117	-75 (-39.1 [-51.6; -23.3])
June	135	156	21 (15.6 [-8.2; 45.5])
July	197	174	-23 (-11.7 [-28.0; 8.3])
August	177	134	-43 (-24.3 [-39.5; -5.2])
September	184	160	-24 (-13.0 [-29.6; 7.5])
October	164	172	8 (4.9 [-15.3; 29.9])
November	165	131	-34 (-20.6 [-36.9; -0.1])
December	164	129	-35 (-21.3 [-37.5; -0.9])
January	197	119	-78 (-39.6 [-51.9; -24.2])
February	185	168	-17 (-9.2 [-26.3; 11.9])
Total	2,095	1,772	-323 (-15.4 [-20.6; -9.9])

Notes: CI, confidence interval.

Supplementary Table 4: Number of carcinoma in situ from March 2019 to February 2021, stratified by tumour site (cervix uteri, bladder, breast, colon, skin/melanoma), period (pre-pandemic: March 2019 to February 2020; pandemic: March 2020 to February 2021), and month of year

	Pre-pandemic period: Cases	Pandemic period: Cases	Difference (% [95% CI])
**Cervix uteri**			
March	81	124	43 (53.1 [15.7; 102.6])
April	79	124	45 (57.0 [18.4; 108.1])
May	100	159	59 (59.0 [23.8; 104.2])
June	55	198	143 (260.0 [167.0; 385.3])
July	80	212	132 (165.0 [104.9; 242.7])
August	70	167	97 (138.6 [80.5; 215.4])
September	102	180	78 (76.5 [38.4; 125.0])
October	101	188	87 (86.1 [46.2; 137.1])
November	82	209	127 (154.9 [97.4; 229.0])
December	102	202	100 (98.0 [56.1; 151.3])
January	92	179	87 (94.6 [51.3; 150.2])
February	98	238	140 (142.9 [91.9; 207.3])
Total	1,042	2,180	1,138 (109.2 [94.3; 125.2])
**Bladder**			
March	91	133	42 (46.2 [11.9; 90.8])
April	78	76	-2 (-2.6 [-29.0; 33.6])
May	87	67	-20 (-23.0 [-44.0; 5.9])
June	72	62	-10 (-13.9 [-38.7; 20.9])
July	99	110	11 (11.1 [-15.3; 45.8])
August	73	84	11 (15.1 [-15.9; 57.5])
September	91	95	4 (4.4 [-21.7; 39.2])
October	117	86	-31 (-26.5 [-44.4; -2.9])
November	83	90	7 (8.4 [-19.5; 46.1])
December	97	88	-9 (-9.3 [-32.0; 21.1])
January	100	87	-13 (-13.0 [-34.7; 16.0])
February	113	103	-10 (-8.8 [-30.2; 19.0])
Total	1,101	1,081	-20 (-1.8 [-9.7; 6.8])
**Breast**			
March	78	66	-12 (-15.4 [-39.0; 17.4])
April	68	58	-10 (-14.7 [-39.9; 21.1])
May	71	36	-35 (-49.3 [-66.0; -24.3])
June	54	51	-3 (-5.6 [-35.6; 38.5])
July	78	65	-13 (-16.7 [-40.0; 15.8])
August	93	52	-41 (-44.1 [-60.2; -21.5])
September	70	60	-10 (-14.3 [-39.3; 21.0])
October	80	81	1 (1.2 [-25.7; 37.9])
November	84	89	5 (6.0 [-21.4; 42.8])
December	73	75	2 (2.7 [-25.6; 41.8])
January	99	72	-27 (-27.3 [-46.3; -1.5])
February	57	66	9 (15.8 [-18.8; 65.0])
Total	905	771	-134 (-14.8 [-22.6; -6.2])
**Colon**			
March	26	17	-9 (-34.6 [-64.5; 20.5])
April	27	14	-13 (-48.1 [-72.8; -1.1])
May	41	20	-21 (-51.2 [-71.4; -16.7])
June	25	7	-18 (-72.0 [-87.9; -35.3])
July	25	17	-8 (-32.0 [-63.3; 25.9])
August	26	13	-13 (-50.0 [-74.3; -2.7])
September	24	12	-12 (-50.0 [-75.0; 0.0])
October	17	20	3 (17.6 [-38.4; 124.6])

November	23	17	-6 (-26.1 [-60.5; 38.3])
December	15	11	-4 (-26.7 [-66.3; 59.7])
January	18	17	-1 (-5.6 [-51.3; 83.3])
February	16	8	-8 (-50.0 [-78.6; 16.8])
Total	283	173	-110 (-38.9 [-49.4; -26.1])
**Skin/Melanoma**			
March	22	21	-1 (-4.5 [-47.5; 73.6])
April	31	17	-14 (-45.2 [-69.6; -0.9])
May	21	11	-10 (-47.6 [-74.7; 8.6])
June	24	15	-9 (-37.5 [-67.2; 19.1])
July	29	20	-9 (-31.0 [-61.0; 21.9])
August	22	16	-6 (-27.3 [-61.8; 38.5])
September	20	24	4 (20.0 [-33.7; 117.2])
October	23	26	3 (13.0 [-35.5; 98.1])
November	25	23	-2 (-8.0 [-47.8; 62.1])
December	22	14	-8 (-36.4 [-67.4; 24.4])
January	17	15	-2 (-11.8 [-55.9; 76.7])
February	24	17	-7 (-29.2 [-61.9; 31.8])
Total	280	219	-61 (-21.8 [-34.5; -6.7])

Notes: CI, confidence interval.

Supplementary Table 5: Number of incident malignant neoplasms (without cervix uteri) and carcinoma in situ (without cervix uteri) from March 2019 to February 2021, stratified by period (pre-pandemic: March 2019 to February 2020; pandemic: March 2020 to February 2021), month of year, and age group

	Pre-pandemic period: Cases	Pandemic period: Cases	Difference (% [95% CI])
**0-49 years**			
March	385	366	-19 (-4.9 [-17.6; 9.7])
April	353	319	-34 (-9.6 [-22.3; 5.1])
May	369	301	-68 (-18.4 [-29.9; -5.0])
June	313	337	24 (7.7 [-7.7; 25.6])
July	419	384	-35 (-8.4 [-20.2; 5.3])
August	353	324	-29 (-8.2 [-21.1; 6.7])
September	357	357	0 (0.0 [-13.6; 15.8])
October	429	392	-37 (-8.6 [-20.3; 4.8])
November	374	355	-19 (-5.1 [-17.9; 9.8])
December	331	340	9 (2.7 [-11.7; 19.5])
January	383	339	-44 (-11.5 [-23.5; 2.4])
February	326	330	4 (1.2 [-13.1; 18.0])
Total	4,392	4,144	-248 (-5.6 [-9.6; -1.6])
**50-69 years**			
March	1,766	1,768	2 (0.1 [-6.3; 6.9])
April	1,678	1,378	-300 (-17.9 [-23.5; -11.8])
May	1,679	1,386	-293 (-17.5 [-23.1; -11.4])
June	1,376	1,538	162 (11.8 [3.9; 20.2])
July	1,943	1,791	-152 (-7.8 [-13.6; -1.7])
August	1,564	1,496	-68 (-4.3 [-10.9; 2.7])
September	1,646	1,654	8 (0.5 [-6.1; 7.6])
October	1,791	1,752	-39 (-2.2 [-8.4; 4.5])
November	1,654	1,721	67 (4.1 [-2.7; 11.3])
December	1,627	1,531	-96 (-5.9 [-12.2; 0.9])
January	1,806	1,322	-484 (-26.8 [-31.8; -21.4])
February	1,696	1,545	-151 (-8.9 [-15.0; -2.4])
Total	20,226	18,882	-1,344 (-6.6 [-8.5; -4.8])
**70 years and above**			
March	1,690	1,707	17 (1.0 [-5.6; 8.0])
April	1,705	1,263	-442 (-25.9 [-31.1; -20.3])
May	1,877	1,426	-451 (-24.0 [-29.1; -18.6])
June	14,42	1,630	188 (13.0 [5.3; 21.3])
July	2,004	2,054	50 (2.5 [-3.6; 9.0])
August	1,616	1,577	-39 (-2.4 [-9.0; 4.6])
September	1,707	1,730	23 (1.3 [-5.2; 8.4])
October	1,839	1,825	-14 (-0.8 [-7.0; 5.9])
November	1,748	1,638	-110 (-6.3 [-12.4; 0.2])
December	1,509	1,394	-115 (-7.6 [-14.1; -0.6])
January	1,866	1,371	-495(-26.5 [-31.5; -21.2])
February	1,811	1,590	-221 (-12.2 [-17.9; -6.1])
Total	20,814	19,205	-1,609 (-7.7 [-9.5; -5.9])

Notes: CI, confidence interval.

Supplementary Table 6: Number of incident malignant neoplasms (without cervix uteri) and carcinoma in situ (without cervix uteri) from March 2019 to February 2021, stratified by period (pre-pandemic: March 2019 to February 2020; pandemic: March 2020 to February 2021), month of year, and sex

	**Pre-pandemic period: Cases**	**Pandemic period: Cases**	**Difference (% [95% CI])**
**Female**			
March	1,899	1,858	-41 (-2.2 [-8.2; 4.3])
April	1,863	1,401	-462 (-24.8 [-29.8; -19.4])
May	1,883	1,476	-407 (-21.6 [-26.8; -16.1])
June	1,456	1,686	230 (15.8 [8.0; 24.2])
July	2,106	2,033	-73 (-3.5 [-9.2; 2.6])
August	1,714	1,676	-38 (-2.2 [-8.6; 4.6])
September	1,778	1,863	85 (4.8 [-1.8; 11.8])
October	1,982	1,938	-44 (-2.2 [-8.2; 4.1])
November	1,830	1,830	0 (0.0 [-6.3; 6.7])
December	1,691	1,681	-10 (-0.6 [-7.1; 6.4])
January	1,903	1,514	-389 (-20.4 [-25.6; -14.9])
February	1,851	1,706	-145 (-7.8 [-13.7; -1.6])
Total	21,956	20,662	-1,294 (-5.9 [-7.7; -4.1])
**Male**			
March	1,942	1,982	40 (2.1 [-4.1; 8.7])
April	1,872	1,559	-313 (-16.7 [-22.1; -10.9])
May	2,041	1,637	-404 (-19.8 [-24.8; -14.4])
June	1,675	1,819	144 (8.6 [1.6; 16.0])
July	2,260	2,195	-65 (-2.9 [-8.4; 3.0])
August	1,818	1,721	-97 (-5.3 [-11.4; 1.1])
September	1,930	1,878	-52 (-2.7 [-8.7; 3.7])
October	2,077	2,031	-46 (-2.2 [-8.0; 4.0])
November	1,945	1,883	-62 (-3.2 [-9.1; 3.1])
December	1,776	1,584	-192 (-10.8 [-16.7; -4.6])
January	2,152	1,517	-635 (-29.5 [-34.0; -24.7])
February	1,982	1,758	-224 (-11.3 [-16.8; -5.4])
Total	23,470	21,564	-1,906 (-8.1 [-9.8; -6.4])

Notes: CI, confidence interval.
